# A Novel XGBoost Method to Infer the Primary Lesion of 20 Solid Tumor Types From Gene Expression Data

**DOI:** 10.3389/fgene.2021.632761

**Published:** 2021-02-03

**Authors:** Sijie Chen, Wenjing Zhou, Jinghui Tu, Jian Li, Bo Wang, Xiaofei Mo, Geng Tian, Kebo Lv, Zhijian Huang

**Affiliations:** ^1^Department of Mathematics, Ocean University of China, Qingdao, China; ^2^Department of Oncology, Hiser Medical Center of Qingdao, Qingdao, China; ^3^Qingdao Geneis Institute of Big Data Mining and Precision Medicine, Qingdao, China; ^4^Geneis Beijing Co., Ltd., Beijing, China; ^5^Department of Breast Surgical Oncology, Fujian Cancer Hospital & Fujian Medical University Cancer Hospital, Fuzhou, China

**Keywords:** tumor tissue-of-origin, gene expression, XGBoost, feature selection, CUP

## Abstract

**Purpose:**

Establish a suitable machine learning model to identify its primary lesions for primary metastatic tumors in an integrated learning approach, making it more accurate to improve primary lesions’ diagnostic efficiency.

**Methods:**

After deleting the features whose expression level is lower than the threshold, we use two methods to perform feature selection and use XGBoost for classification. After the optimal model is selected through 10-fold cross-validation, it is verified on an independent test set.

**Results:**

Selecting features with around 800 genes for training, the *R*^2^-score of a 10-fold CV of training data can reach 96.38%, and the *R*^2^-score of test data can reach 83.3%.

**Conclusion:**

These findings suggest that by combining tumor data with machine learning methods, each cancer has its corresponding classification accuracy, which can be used to predict primary metastatic tumors’ location. The machine-learning-based method can be used as an orthogonal diagnostic method to judge the machine learning model processing and clinical actual pathological conditions.

## Introduction

Metastatic cancer is a metastatic malignant tumor that has been confirmed by biopsy, but the primary site cannot be found. The cancer cells from the primary site are brought into other organs by invading the lymph, blood, or other means (Pavlidis and Pentheroudakis, 2012). The cause of the tumor is that the focus is small, the position is hidden, or the site of the disease is in the lower part of the mucous membrane and the like, the focus is not easy to find, and the biological behavior of the tumor is worse, leading to the early metastasis of the tumor (Smith et al., 1967).

It is particularly important to find the primary focus in the clinical stage of cancer treatment. Only by finding the primary focus can the clinical cure rate of the patient be improved. Because the biological features often vary with the type of tumor tissue, we can make a pathological diagnosis based on the existing biological knowledge and established pathological methods. Due to the limited tissue and diagnostic staining of tumors and the influence of doctors’ professional level, there are still some loopholes and shortcomings in the thorough search at this stage (Medeiros et al., 2010; Eti et al., 2012; Angela et al., 2017).

The transfer of cancer means that the tumor cells are taken to it from the primary site into the lymphatic vessel, the blood vessel, or other means to continue to grow to form the same type of tumor as the primary site. Common methods of transfer include lymphatic metastasis, vascular metastasis, and the like. About 50% of the lung cancer will have multiple bone metastasis sites, 28–33% of the liver metastasis, and 17–20% of the transfer of the kidney and the epinephrine. The auxiliary imaging examination is usually diagnosed by a biochemical indicator. In the liver metastases, the biochemical biopsy of the liver micro metastases may cause confusion due to the stability of the biochemical indicators; and in the imaging ultrasound examination, the lesions of 1–2 cm could be detected in random tests. The error of uncertain factors in a practical application will accumulate and magnify, resulting in diagnostic confusion.

We aim to establish an automatic processing method to solve this problem. We selected data from gene expression profiles. By analyzing and processing the existing data, a relatively suitable machine learning model is obtained (Fei et al., 2020), and the efficiency of diagnosis of primary lesions can be improved to be more accurate. Different tumorous types have distinct expression profiles on specific genes, and the difference could be captured by the machine learning models and used to classify the primary lesions.

In essence, machine learning trains computers to simulate or realize human learning behavior to acquire new knowledge and skills and reorganize the existing knowledge structure to improve its own performance continuously. The application of medical treatment is also a process of comprehensive doctor diagnosis experience to treat patients. Many machine learning algorithms have been developed for classification problems. It can judge the unknown information by learning from the known information. By studying the existing tumor samples’ features, the computer has a certain decision-making ability to judge and evaluate the unknown cancer pathology directly.

XGBoost based on tree boosting is a scalable end-to-end tree boosting system, which was first proposed by Chen and Guestrin (2016). This system is an open-source system available at https://github.com/dmlc/xgboost and is widely used in bioinformatics. Mendik et al. (2018) use XGBoost for analyzing protein translocation between cellular organelles; Li et al. (2019) use XGBoost for predicting gene expression values; Danciu et al. (2020) use XGBoost for predicting early-stage prostate cancer in veterans. We describe the algorithm mechanism in detail in the methods section.

## Materials and Methods

### Data Preparation

#### Training Set and Oversampling

Data of 5,759 samples, each containing 20,501 gene characteristics, were downloaded from TCGA. After extracting effective information, we normalized the gene expression by the sum of all the sample gene expressions. We use oversampling with stable results to solve the problem of data imbalance, then we select and train the optimal model 10-fold cross-validation on TCGA data.

#### Test Set

We conduct retrospective testing on a GEO test set containing 42 samples covering five cancers. The trained model predicts the test data, and the results were compared with the true labels of the samples. The specific number of samples per cancer is shown in Table 1.

**TABLE 1:** Data size and proportion.

Training data from TCGA		
Cancer type	Amount	Percent
BRCA	1,056	0.13687622
KIRC	526	0.06817887
UCEC	516	0.0668827
THCA	500	0.06480881
LUAD	486	0.06299417
HNSC	480	0.06221646
COAD	451	0.05845755
LGG	439	0.05690214
STAD	415	0.05379132
PRAD	379	0.04912508
BLCA	301	0.03901491
LIHC	294	0.03810758
OV	261	0.0338302
CESC	258	0.03344135
KIRP	222	0.02877511
LAML	173	0.02242385
GBM	153	0.0198315
READ	153	0.0198315
PAAD	142	0.0184057
SKCM	80	0.01036941
Unknown	430	0.05573558
**Testing data from GEO**		
BRCA	13	0.27659574
COADREAD	2	0.04255319
LIHC	5	0.10638298
LUAD	15	0.31914894
OV	12	0.25531915

### Feature Selection Method

In the training set and the independent verification set, a part of the gene expression level was very low. We set the expression level threshold value as 0.00005, 0.00001, and 0.000001, respectively, for screening. After the intersection of the training set’s gene characteristics and the independent verification set, the following feature selection was conducted.

We choose the Chi-Square test and Random Forest in the filtering method for feature selection. The Chi-Square calculates the correlation of qualitative independent variables to qualitative dependent variables. First, we take each gene as an independent hypothesis and then calculate the degree of deviation D between the observed value and the theoretical value. If the deviation is small enough, accept the null hypothesis; otherwise, reject the null hypothesis, and accept the alternative hypothesis. Therefore, the larger the deviation value D, the greater the deviation from the original hypothesis. That is, the more relevant it is, the better the selection process becomes at calculating the deviation value D of each gene and the type of cancer, and to order them from large to small, and to take the first *k* genes.

The application of random forest in feature selection needs to calculate the feature importance. The specific steps are as follows: First, we calculate each feature’s importance and sort it in descending order. After that, we determine the proportion to be eliminated and get a new feature set by eliminating the corresponding proportion of features according to their importance. Repeat the process with the new feature set until there are m features left, which is the preset value. Finally, we select the feature set with the lowest out-of-bag error rate according to each feature set obtained in the above process and the corresponding out-of-bag error rate of the feature set.

### Training Method

XGBoost is based on gradient tree boosting. Unlike traditional trees, which only do the first-order Taylor expansion, XGBoost performs the second-order Taylor expansion, which realizes the parallel computation (Li et al., 2019). It can use the combination of weak learners to create a single strong learner to reach a fast execution speed and a good model performance. Its main idea is to continuously add a tree and continuously perform feature splitting to grow a tree. Each time a tree is added, it is learning a new function to fit the last prediction residuals. If we get *k*-trees after training, we need to predict the score of a sample. In fact, according to the characteristics of this sample, each tree will fall to a corresponding leaf node, and each leaf node corresponds to a score. It is necessary to add up the scores corresponding to each tree to be the predicted value of the sample. Chen and Guestrin (2016) descript the mathematical formula of gradient tree boost and XGBoost with scientific rigor. And Li et al. (2019) described the parameters of XGBoost.

We fine-tuned three hyperparameters within the 10-fold cross-validation. The parameter “n estimators” is the number of trees to be used in the forest. The parameter “max depth” is the deepest depth of all trees. The parameter “min child weight parameter” in XGBoost is the minimum sum of instance weight (hessian) needed in a child. If the tree partition step results in a leaf node with the sum of instances weighing less than the min child weight, the building process will give up further partitioning. This parameter is used to avoid overfitting. When its value is large, the model can be prevented from learning from outliers. But if this value is too high, it will cause under-fitting. The max depth is also used to avoid overfitting. The greater the max depth, the more outliers the model will learn.

### Parameters of Model Evaluation and Parameters in the Results

Use the *R*^2^ score as an indicator of the evaluation model. At the same time, the test results are output, which included the *R*^2^ score, precision, recall rate, and the F1 score of each cancer calculation result shown in Table 2.

**TABLE 2:** Parameters of model evaluation and parameters in the results.

R^2^ score	1-MSE(ŷ,y)/Var(y)	
Precision	TP / (TP+FP)	
Recall rate	TP / (TP+FN)	
F1score	⋅(Precision⋅ Recall)/(Precision+Recall)	
	
	**Relevant**	**No relevant**

**Retrieved**	True positives (TP)	False positives (FP)
**Not retrieved**	False negatives (FN)	True negatives (TN)
Precision TP / (TP + FP) Recall rate TP / (TP + FN) F-Score=(1+β^2^)⋅ (Precision⋅ Recall)/(β^2^⋅ Precision+Recall)		

The predicted value is $\hat{y}$ and the true value is y. *R*^2^ score the problem that MSE (Mean Absolute Error), RMSE (Root Mean Squared Error), and MAE (Mean Absolute Error) cannot solve when dimensions are different, and it is difficult to measure the effectiveness of the model. *R*^2^ score = 1, reaches the maximum value, and then MSE as the molecule is 0, which means that the predicted value and the true value in the sample are the same, without any error. In other words, the model that has been established perfectly fits all the real data, which is the model with the best effect and where the *R*^2^ score value reaches the maximum. The model is usually not so perfect; there are always errors; when the error is small, the numerator is less than the denominator; when the model tends to 1, it is still a good model. Precision is defined as (true-positives)/(true positives + false- positives). Recall rate is defined as (true-positives)/(true-positives + false-negatives), which intuitively represents the classifier’s ability to identify all positive cases correctly. F1 score is the harmonic mean of precision and recall. Precision and Recall do not have much of a relationship with the formula, but they are mutually restricted in practice. We all hope that the model is accurate, and the recall rate is high, but when the precision rate is high, the recall rate is often low. When β = 1, it becomes the F1-score, in which case both recall, and accuracy are important and have the same weight. In some cases, if we think accuracy is more important, we adjust the β value to be less than 1, and if we think the recall is more important, we adjust the β value to be greater than 1, such as the F2-score.

We determined the data list as the first 800 genes from the feature selection list. We used software: Cytoscape and metascape for GO (Gene Ontology) and KEGG (Kyoto Encyclopedia of Gene and Genomes) Enrichment Analysis.

## Results

### Genes Selected by Random Forest Were More Informative Than Chi-Square

**FIGURE 1:**
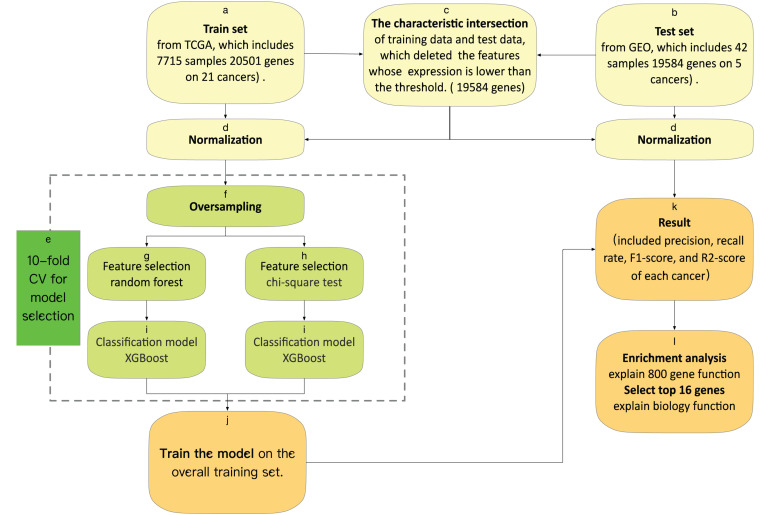
(a) The training set was downloaded from TCGA, which obtains 5,759 samples, each containing 20,501 gene characteristics on 21 cancers. (b) The test set was downloaded from GEO, which obtains 42 samples, each containing 19,584 gene characteristics on five cancers. (c) The characteristic intersection of training data and test data deletes the features whose expression is lower than the threshold. (d) We normalize the gene expression by the sum of all the gene expressions in each sample. (e) Model selection by the result of 10-fold cross-validation. (f) We oversample the train set, and then we have data for 854 samples of each tumor. (j) We train the model on the overall training set, with optimal features and an optimal model. (k) We test the model and output the result included precision, recall rate, F1-score, and *R*^2^-score of each cancer. (l) We do an enrichment analysis of the top 800 genes with KEGG (Kyoto Encyclopedia of Genes and Genomes) and GO (Gene Ontology) by metascape, and explain the top 16 genes from feature selection, which include biological function.

We used 10-fold cross-validation in the training set to evaluate the performance of the feature selection methods. With leave-one-out cross-validation, the algorithm is repeatedly retrained, which included oversampling, feature selection, and classification model, leaving out one sample in each round and testing each sample on a classifier that was trained without this sample. The framework of the 10-fold CV is shown in Figure 1.

**FIGURE 2:**
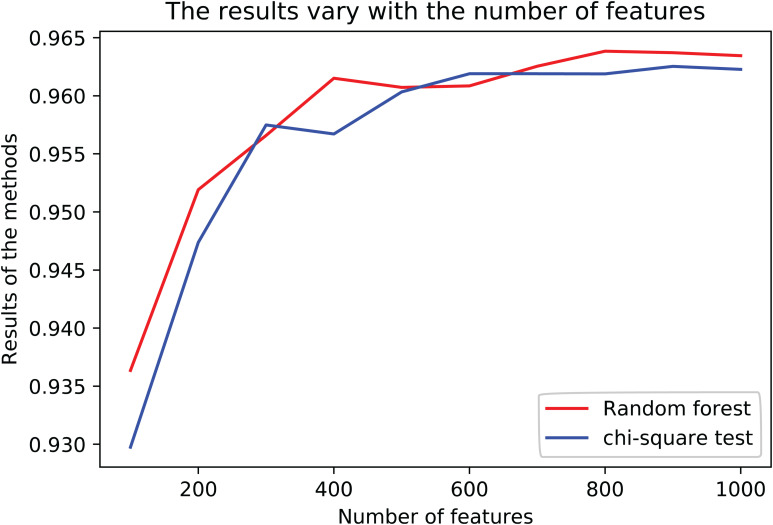
The results of variety with the number of features in different methods.

The results are shown in Table 3. The average *R*^2^-score of 10-fold cross-validation of the two feature selection methods is very high. The average *R*^2^-score was 96.23 and 96.38% (95% confidence interval) for the chi-square test as feature selection and random forest as feature selection. Although these two results are very close, the *R*^2^-score of Random Forest is slightly higher than the Chi-Square within the same feature number range, and the Rise of *R*^2^-score of random forest is more stable, as shown in Figure 2. Considering all the results of the average *R*^2^-score, the Random Forest is used for feature selection in the next flow.

**TABLE 3:** 10-fold CV results of variety with the number of features in Chi-Square and Random Forest.

Feature number	10-fold CV result of using the Chi-Square in feature selection	10-fold CV result of using Random Forest in feature selection
100	0.929750576	0.936357298
200	0.947377573	0.951911924
300	0.957487752	0.956577824
400	0.956709878	0.961505816
500	0.960339005	0.960726262
600	0.961894081	0.960854956
700	0.961894081	0.962541414
800	0.961890889	**0.963838431**
900	0.962538726	0.963707385
1,000	**0.962278986**	0.963448150

*The bold values in each column are the optimal results for this method.*

### The XGBoost Algorithm Showed Good Generalization Performance on the GEO Dataset

We selected 800 genes with Random Forest characteristics, using XGBoost as a classifier. Taking the *R*^2^-score as the model evaluation index, 10-fold CV was carried out in the training data, and finally, the parameters, n estimators = 250, max depth = 7, min child weight = 1, in the optimal model of XGBoost were obtained. The results of this model in leaving out one data are shown in Figure 2.

**FIGURE 3:**
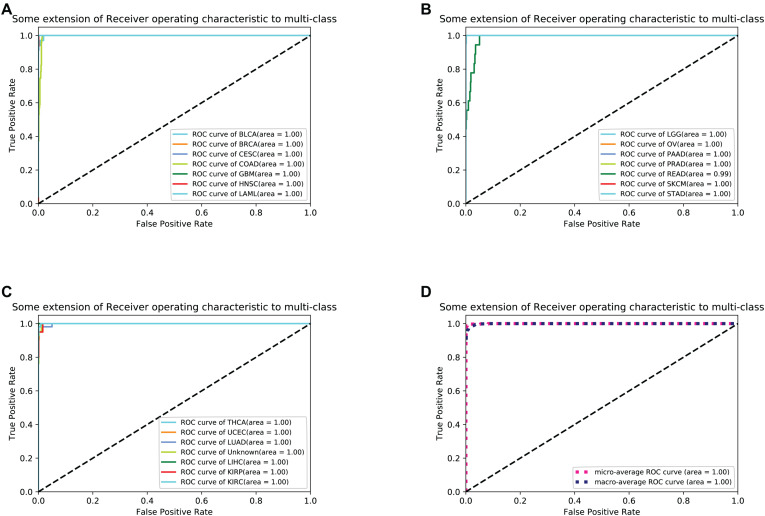
**(A–C)** shows 21 cancers’ ROC curve of the optimal 10-fold CV’s results. **(D)** shows the average ROC curve.

For each sample, the type of tumor predicted was compared with the type diagnosed. When the predicted tumor type matches the reference diagnosis, it is a true positive. When the predicted tumor type does not match the diagnosis, the sample is considered a false-positive. For each cancer, sensitivity was defined as the ratio of true positive results to the total positive samples analyzed, and specificity was defined as the ratio of (1- false positive) to (total test results - total positive). To better measure the classification results, we took sensitivity and specificity as the horizontal axis and the vertical axis, respectively, and drew the ROC (Receiver Operating Characteristic) curve to the results as shown in Figure 3.

**FIGURE 4:**
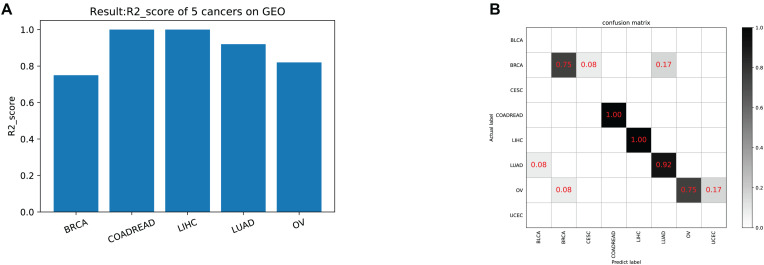
**(A)** The model test result (*R*^2^-score) on five cancers on GEO. **(B)** The confusion matrix on testing data. Our trainer contained 21 cancer tags, but only five cancers in the test set. There was a partial error in the classifier’s prediction outside of the five cancers.

The model was trained according to N estimators = 250, Max depth = 7, and min child weight = 1 in the whole training data for independent testing. The *R*^2^-score average of independent testing results is 83.3%, which obtained 42 samples cover five cancers. The trainer had good generalization for COADREAD (Colon Adenocarcinoma and Rectum Adenocarcinoma), LIHC (Liver Hepatocellular Carcinoma), LUAD (Lung Adenocarcinoma), and OV (Ovarian Serous Cystadenocarcinoma), and the *R*^2^-score respectively was 1, 1, 0.92 and 0.82, shown in Table 4 and Figure 4A. For BRCA (Breast Invasive Carcinoma), we can see from Figure 4B that it is often incorrectly predicted for CESC (Csquamous Cell Carcinoma and Endocervical Adenocarcinoma) and LUAD.

**TABLE 4:** The model test result (precision, recall, F1-score, and *R*^2^-score) on 9 cancers on the GEO dataset.

Abbreviation	Precision	Recall	F1-score	*R*^2^-score	Support
BRCA	1	0.75	0.86	0.75	12
COADREAD	1	1	1	1	1
LIHC	1	1	1	1	5
LUAD	0.85	0.92	0.88	0.92	12
OV	1	0.82	0.9	0.82	11
Avg/total	0.93	0.83	0.87	0.83	42

### Top 16 Genes on Feature Selection

**FIGURE 5:**
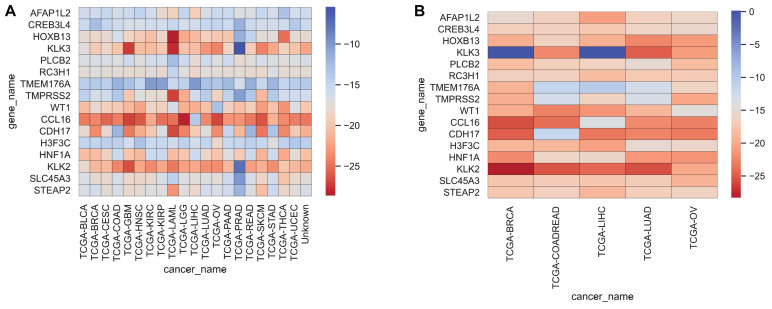
Heatmap representing the expressions of 16 genes for each cancer sample in the training set and test set, averaged, and then logarithmic. Cool colors represent a higher expression level, and warm colors a lower expression level. **(A,B)** Represent the expression levels of 16 genes in the training set and the test set respectively.

We often use molecular experiments to distinguish the origin of metastatic cancer. Our supporting results combined with the literature review found that the accuracy of cancer classification was low for fixed cancer types, which was similar to other data methods. We selected 16 genes, shown in Table 5, with high expression levels, to analyze the potential relationship between these genes and cancer. The heat maps of the expressions of 16 genes in the training set and the test set are shown in Figure 5.

**TABLE 5:** The basic information of top 16 genes on feature selection.

Mark rank	Gene symbol	Gene name	RefSeq DNA sequence	UniProtKB/Swiss-Prot
1	AFAP1L2	Actin filament associated protein 1 like 2	NC_000010.11	Q8N4 × 5-AF1L2_HUMAN
2	CREB3L4	CAMP responsive element binding protein 3 like 4	NC_000001.11	Q8TEY5-CR3L4_HUMAN
3	HOXB13	Homeobox B13	NC_000017.11	Q92826-HXB13_HUMAN
4	KLK3	Kallikrein related peptidase 3	NC_000019.10	P07288-KLK3_HUMAN
5	PLCB2	Phospholipase C beta 2	NC_000015.10	Q00722-PLCB2_HUMAN
6	RC3H1	Ring finger and CCCH-type domains 1	NC_000001.11	Q5TC82-RC3H1_HUMAN
**7**	TMEM176A	Transmembrane protein 176A	NC_000007.14	Q96HP8-T176A_HUMAN
8	TMPRSS2	Transmembrane serine protease 2	NC_000021.9	O15393-TMPS2_HUMAN
9	WT1	WT1 transcription factor	NC_000011.10	P19544-WT1_HUMAN
10	CCL16	C-C motif chemokine ligand 16	NC_000017.11 NT_187614.1	O15467-CCL16_HUMAN
11	CDH17	Cadherin 17	NC_000008.11	Q12864-CAD17_HUMAN
12	H3F3C	Histone variant H3.5	NC_000012.12	Q6NXT2-H3C_HUMAN
13	HNF1A	HNF1 homeobox A	NC_000012.12	P20823-HNF1A_HUMAN
14	KLK2	Kallikrein related peptidase 2	NC_000019.10	P20151-KLK2_HUMAN
15	SLC45A3	Solute carrier family 45 member 3	NC_000001.11	Q96JT2-S45A3_HUMAN
16	STEAP2	STEAP2 metalloreductase	NC_000007.14	Q8NFT2-STEA2_HUMAN

Genes control protein expression. A gene contains introns and exons, in which the coding region of the protein is encoded. Gene coding of a protein is a DNA-mRNA- protein process. The genes we analyzed are all protein-coding genes.

WT1 is a tumor suppressor gene associated with the development of a Wilms’ Tumor, for which it was named. This gene encodes a transcription factor that contains four zinc-finger motifs at the C-terminus and a proline/glutamine-rich DNA-binding domain at the N-terminus. CCL16 is one of several cytokine genes clustered on the q-arm of chromosome 17. Cytokines are a family of secreted proteins involved in immunoregulatory and inflammatory processes. The CC cytokines are proteins characterized by two adjacent cysteines. The cytokine encoded by this gene displays chemotactic activity for lymphocytes and monocytes but not for neutrophils. This cytokine also shows a potent myelosuppressive activity and suppresses the proliferation of myeloid progenitor cells. The expression of this gene is upregulated by IL-10. The CDH17 gene is a member of the cadherin superfamily, genes encoding calcium-dependent, membrane-associated glycoproteins. Diseases associated with CDH17 include Metanephric Adenoma and Cleft Lip/Palate-Ectodermal Dysplasia Syndrome, which is provided by RefSeq et al. Histones are basic nuclear proteins that are responsible for the nucleosome structure of the chromosomal fiber in eukaryotes. Nucleosomes consist of approximately 146 bp of DNA wrapped around a histone octamer composed of pairs of each of the four core histones (H2A, H2B, H3, and H4). Among its related pathways are Transcriptional misregulation in cancer and Activated PKN1, which stimulates transcription of AR (androgen receptor) regulated genes KLK2 and KLK3. HNF1A encodes a transcription factor required for the expression of several liver-specific genes. Diseases associated with HNF1A include Maturity-Onset Diabetes of the Young, Type 3 and Diabetes Mellitus, and Insulin-Dependent 20.

### Enrichment Analysis

**FIGURE 6:**
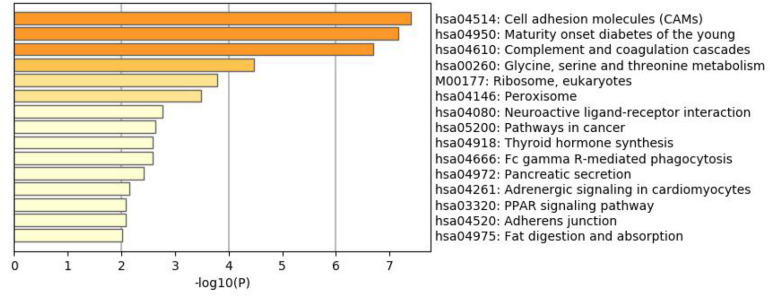
KEGG enrichment analysis of the 800 selected genes.

**FIGURE 7:**
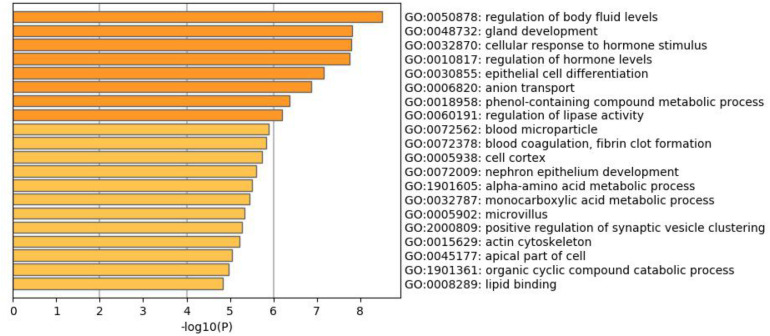
GO enrichment analysis of the 800 selected genes.

**FIGURE 8:**
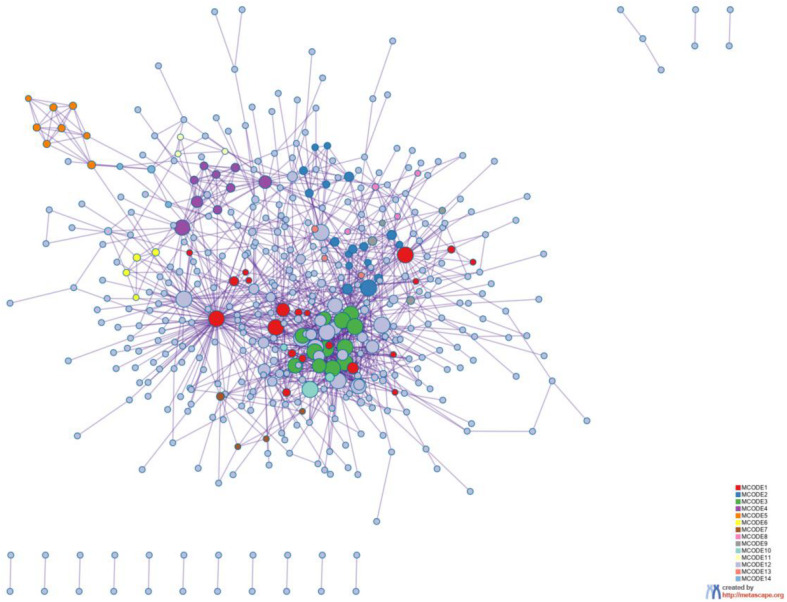
Protein-protein interaction network. The MCODE algorithm was then applied to this network to identify neighborhoods where proteins are densely connected. Each MCODE network is assigned a unique color. The GO enrichment analysis was applied to each MCODE network to assign “meanings” to the network component.

**FIGURE 9:**
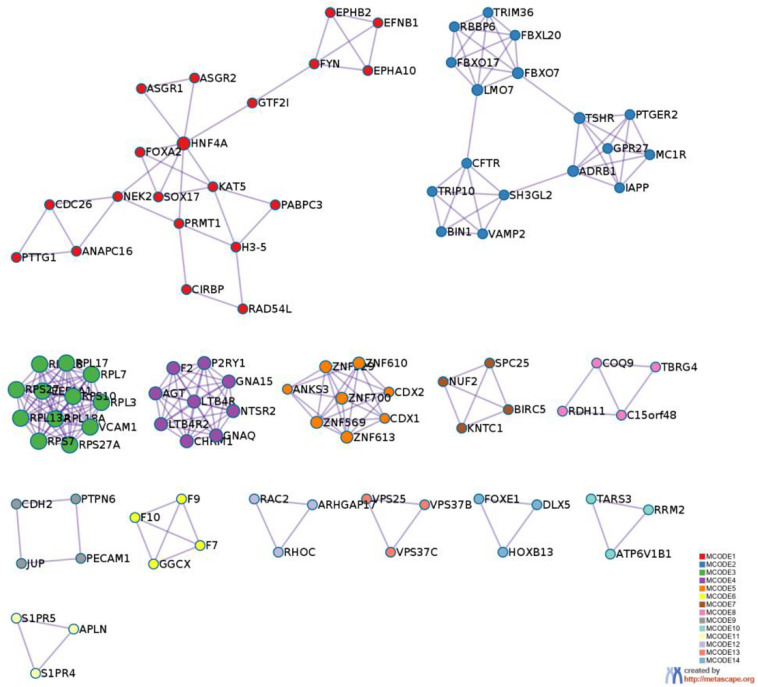
PPI MCODE components.

To better understand why those genes could tell the origin of the primary lesion, we performed the enrichment analysis using the 800 selected genes. The results of KEGG (Kyoto Encyclopedia of Gene and Genomes) (Figure 6) and GO (Gene Ontology) (Figure 7) are shown in Figures 8, 9.

The 800 selected genes were significantly enriched in some cancer-related pathways. Cell adhesion molecules (CAM) (Okegawa et al., 2004) played important roles in invasive and metastasis and cancer progression. Loss of the tumor cells’ intercellular adhesion might result in cells escaping from the primary lesion and metastasizing. CAM is also involved in various functions such as cell growth, differentiation, site-specific gene expression, and morphogenesis, which could explain why the different tissues have different expression profiles among those genes.

The 800 genes were also significantly enriched in some organ-specific pathways. The selected genes were representative in thyroid hormone synthesis, pancreatic secretion, and fat digestion—absorption pathways. Since those pathways were organ-specific, we could show that the random forest algorithm found the differentially expressed genes among different organs.

## Discussion

Nowadays, CUP cases are characterized by small primary tumors (difficult to be detected by existing technologies) (Hainsworth and Greco, 2018), primary tumors being eliminated by the body’s autoimmune system, and primary tumors being excised during surgery (without histological examination), which makes it difficult to find the primary tumors, leading to generally poor prognosis of patients treated with chemotherapy. Our study hopes to help doctors clinically identify the primary of CUP and to use more effective targeted therapies for CUP patients according to these identification results.

In this paper, we show that our result is better than in recent studies. Our average *R*^2^-score of the classification based on XGBoost can reach 96.38%, while the average accuracy of the support vector machine (SVM) classifier is 82–89% (Tothill et al., 2005; Ma et al., 2006). We train a classifier, selected feature by random forests, classified by XGBoost, on data containing 7,715 samples and 19,854 genes from TCGA, and test it on data including 42 samples and five cancers. Currently, the prediction for CUP cancer is between 80%–95% (Sarah, 2010; Greco et al., 2012; Meiri et al., 2012; Conway et al., 2019), and this data fluctuation is related to the different evaluation indicators and sample types of each model. In the test *R*^2^-score of 83.3% in particular, our classifier was relatively accurate in predicting LIHC (liver hepatocellular carcinoma) which is, LUAD (lung adenocarcinoma), OV (ovarian serous cystadenocarcinoma).

Although we have made progress in these studies, there are also limitations. Our test data are collected from 8 series, and there was some detection method between each series. This may be due to the fact that our test results are not as high as the cross-validation results.

Further studies could be done in several main aspects. First, the SNP (single nucleotide polymorphism) or methylation data may be combined with expression profiles to further improve the prediction utilities to infer primary lesions for metastatic tumors. Second, the eQTL (expression Quantitative Trait Loci), which supplies us with new insights between expression profile and mutation profile, might also help determine the primary lesions.

## Conclusion

These findings suggest that by combining multiple tumor data with machine learning methods, each cancer has its corresponding classification accuracy, which can be used to predict primary metastatic tumors’ location. At the same time, it can also be used as an orthogonal diagnostic method to utilize the machine learning model processing for auxiliary diagnosis methods.

## Data Availability Statement

The original contributions presented in the study are included in the article/supplementary material, further inquiries can be directed to the corresponding author/s.

## Author Contributions

KL and ZH designed the study. SC, WZ, JT, and BW collected the data, analyzed the data, interpreted the data. SC wrote the manuscript. JL, XM, and GT reviewed the manuscript. All authors contributed to the article and approved the submitted version.

## Conflict of Interest

BW, XM, and GT were employed by the company Geneis Beijing Co., Ltd. The remaining authors declare that the research was conducted in the absence of any commercial or financial relationships that could be construed as a potential conflict of interest.
